# Altered Structural and Functional Patterns Within Executive Control Network Distinguish Frontal Glioma-Related Epilepsy

**DOI:** 10.3389/fnins.2022.916771

**Published:** 2022-05-26

**Authors:** Guangfu Di, Mingze Tan, Rui Xu, Wei Zhou, Kaiqiang Duan, Zongwen Hu, Xiaoxiang Cao, Hongchuang Zhang, Xiaochun Jiang

**Affiliations:** ^1^Department of Neurosurgery, The Translational Research Institute for Neurological Disorders of Wannan Medical College, The First Affiliated Hospital of Wannan Medical College, Yijishan Hospital of Wannan Medical College, Wuhu, China; ^2^Department of Radiology, The First Affiliated Hospital of Wannan Medical College, Yijishan Hospital of Wannan Medical College, Wuhu, China

**Keywords:** epileptic seizure, executive control network, functional connectivity, gray matter, frontal glioma, pattern classification, regional homogeneity

## Abstract

**Background:**

The tumor invasion of the frontal lobe induces changes in the executive control network (ECN). It remains unclear whether epileptic seizures in frontal glioma patients exacerbate the structural and functional alterations within the ECN, and whether these changes can be used to identify glioma-related seizures at an early stage. This study aimed to investigate the altered structural and functional patterns of ECN in frontal gliomas without epilepsy (non-FGep) and frontal gliomas with epilepsy (FGep) and to evaluate whether the patterns can accurately distinguish glioma-related epilepsy.

**Methods:**

We measured gray matter (GM) volume, regional homogeneity (ReHo), and functional connectivity (FC) within the ECN to identify the structural and functional changes in 50 patients with frontal gliomas (29 non-FGep and 21 FGep) and 39 healthy controls (CN). We assessed the relationships between the structural and functional changes and cognitive function using partial correlation analysis. Finally, we applied a pattern classification approach to test whether structural and functional abnormalities within the ECN can distinguish non-FGep and FGep from CN subjects.

**Results:**

Within the ECN, non-FGep and FGep showed increased local structure (GM) and function (ReHo), and decreased FC between brain regions compared to CN. Also, non-FGep and FGep showed differential patterns of structural and functional abnormalities within the ECN, and these abnormalities are more severe in FGep than in non-FGep. Lastly, FC between the right superior frontal gyrus and right dorsolateral prefrontal cortex was positively correlated with episodic memory scores in non-FGep and FGep. In particular, the support vector machine (SVM) classifier based on structural and functional abnormalities within ECN could accurately distinguish non-FGep and FGep from CN, and FGep from non-FGep on an individual basis with very high accuracy, area under the curve (AUC), sensitivity, and specificity.

**Conclusion:**

Tumor invasion of the frontal lobe induces local structural and functional reorganization within the ECN, exacerbated by the accompanying epileptic seizures. The ECN abnormalities can accurately distinguish the presence or absence of epileptic seizures in frontal glioma patients. These findings suggest that differential ECN patterns can assist in the early identification and intervention of epileptic seizures in frontal glioma patients.

## Introduction

Glioma is the most common malignant tumor of the central nervous system (Ostrom et al., 2019), and the incidence of epilepsy in glioma can be as high as 40–90% (Pallud et al., 2014; Fisher et al., 2017; Akeret et al., 2020; You et al., 2020). Tumor-related epilepsy profoundly impacts patients’ prognosis and quality of life (Maschio and Dinapoli, 2012). However, how tumors induce epilepsy is still unclear. This poses a significant challenge for antiepileptic therapy in patients with newly diagnosed tumors (Siomin et al., 2005; Tremont-Lukats et al., 2008). Tumor invasion of the frontal lobe induces changes in the executive control network (ECN) (Liu Y. et al., 2020). Similarly, the onset of epilepsy also leads to functional network alterations (Cao et al., 2014; Fang et al., 2022). Therefore, an in-depth understanding of the structural and functional patterns of the ECN in glioma-associated epilepsy is required for the early differentiation and diagnosis of glioma seizures and the development of individualized intervention strategies.

Executive control network is a frontoparietal circuit involved in executive functions (Crone et al., 2006; Sakai and Passingham, 2006; Dosenbach et al., 2007; Seeley et al., 2007, 2009; Brier et al., 2012; Chen et al., 2016b). Tumor invasion of the frontal lobe (located in ECN) affects the brain’s executive functions (Seeley et al., 2009; Smith et al., 2009; Park et al., 2015; Zhang et al., 2016; Liu et al., 2019). Recent studies have consistently indicated that when the tumor invades the frontal lobe, the frontal glioma shows structural [gray matter (GM) reorganization and reduction in cortical thickness] (Kinno et al., 2020; Liu Y. et al., 2020) and functional [functional reorganization and decreased functional connectivity (FC)] abnormalities within the ECN (Liu et al., 2019; Liu Y. et al., 2020; Tordjman et al., 2021). Furthermore, idiopathic epilepsy reduces FC in networks (such as the salience network, default network, and ECN) (Cao et al., 2014; Li et al., 2017). In particular, some studies indicate that tumor-related epilepsy induces topological changes in white matter (WM) networks (Zhou et al., 2022) and functional networks (Fang et al., 2022). Based on these findings, we hypothesize that tumor invasion of the frontal lobe, accompanied by epileptic seizures, will lead to disruption and reorganization of the ECN. This damage will be aggravated compared to glioma patients without epilepsy.

In glioma surgery, after tumor control, treatment of glioma-related epilepsy is the second primary purpose (You et al., 2020). When combined with preoperative individualized and standardized antiepileptic therapies, glioma surgery can be highly effective in epilepsy control (You et al., 2020). However, identifying epileptic seizures early in glioma and administering individualized anti-seizure medicine is a significant challenge in clinical practice. As the clinical importance of radiomics and artificial intelligence (AI) has increased in recent years, deep learning and machine learning (ML) combined with multimodal imaging features have been widely used to predict glioma grades (Vamvakas et al., 2019; Abdelaziz Ismael et al., 2020), survival (Sanghani et al., 2018; Peeken et al., 2019; Choi et al., 2020), and molecular phenotype (Tan et al., 2019; Bangalore Yogananda et al., 2020). Furthermore, AI-based ML methods have been applied to various areas of epilepsy research, such as structural connectome and diffusion tensor imaging features (Munsell et al., 2015; Gleichgerrcht et al., 2018; Abbasi and Goldenholz, 2019). With this knowledge, we theorize that the combination of ML and multimodal neuroimaging data may be helpful in the early identification of glioma seizures. In particular, the structural and functional abnormalities of the ECN may be used for the early identification of frontal glioma-associated epileptic seizures.

This study aimed to investigate the altered structural and functional patterns of ECN in non-FGep and FGep subjects and to evaluate whether these patterns can accurately distinguish glioma-associated epileptic seizures. Based above-mentions studies (Liu et al., 2019; Liu Y. et al., 2020; Tordjman et al., 2021), we hypothesized that non-FGep and FGep would show structural (i.e., GM) and functional [i.e., regional homogeneity (ReHo) and FC] abnormalities of ECN and display differential damage patterns. Furthermore, we hypothesize that the altered patterns can accurately distinguish tumor invasion of the frontal lobe with or without an epileptic seizure. To test the hypothesis, we identified structural and functional changes within the ECN in non-FGep and FGep patients. Next, we applied a pattern classification approach to test how well structural and functional abnormalities within ECN could distinguish non-FGep and FGep from CN subjects.

## Materials and Methods

### Subjects

We recruited 50 patients with frontal gliomas from inpatients at the Department of Neurosurgery, Yijishan Hospital of Wannan Medical College (China). Patients were divided into 29 non-FGep (no clear history of seizures) and 21 FGep (clear history of seizures). The diagnosis of glioma-related epilepsy was based on clinical markers and electroencephalography findings. The CN were recruited through advertisements and matched to patients with frontal gliomas according to age, gender, and education (Table 1). The subjects signed written informed consent forms approved by the Human Participants Ethics Committee of Yijishan Hospital of Wannan Medical College (China).

**TABLE 1:** Demographic and cognitive measures of CN, non-FGep, and FGep subjects.

Items	CN (*n* = 39)	Non-FGep (*n* = 29)	FGep (*n* = 21)
Age (years)	53.49 (5.79)	53.34 (13.20)	53.05 (14.98)
Gender (M/F)	18/21	17/12	12/9
Education level (years)	11.97 (2.52)	11.93 (2.77)	11.57 (2.91)
**Cognitive function**			
AVLT	0.23 (0.80)	0.30 (0.84)	−0.83 (1.13)^a,b^
DST	0.25 (1.24)	−0.22 (0.69)	−0.17 (0.76)
TMT	0.46 (1.00)	−0.41 (0.54)^a^	−0.28 (1.17)^a^
CDT	0.11 (0.88)	0.03 (1.13)	−0.17 (0.76)

*Values are expressed as the mean (standard deviation, SD). ^a^Significant differences were found between CN and non-FGep, CN, and FGep, ^b^Significant differences were found between non-FGep and FGep. Raw cognitive scores were converted to Z scores based on the mean and SD of the raw scores for three group subjects. Notably, for TMT measured by timing, the raw scores of TMT were defined as the reciprocal of the time required for the test. CN, healthy controls; non-FGep, frontal glioma without epilepsy; FGep, frontal glioma with epilepsy; AVLT, Auditory Verbal Learning Test – 20-min delayed recall; DST, Digit Span Test; TMT, Trail Making Test; CDT, Clock Drawing Test.*

The inclusion criteria for the patient groups were as follows: (1) tumor pathology was confirmed as primary glioma by surgery, (2) the tumor invaded the frontal lobe, (3) the extension of the tumor had not reached the central sulcus, (4) glioma had unilateral tumor invasion, (5) glioma had no brain injury, and (6) no history of biopsy, radiotherapy, or chemotherapy. The exclusion criteria were: (1) multiple lesion foci, (2) history of substance abuse, (3) magnetic resonance imaging (MRI) contraindications, and (4) gliomas involving bilateral prefrontal lobes.

### Neuropsychological Assessments

A neuropsychological battery was utilized to assess episodic memory (auditory verbal learning test- 20-min delay recall, AVLT-20-DR), information processing speed (trail making test-A, TMT), visuospatial function (clock drawing test, CDT), and executive function (digit span test, DST). All subjects underwent a standardized clinical interview and comprehensive neuropsychological assessments that were evaluated by two neuropsychologists. These are senior doctors with more than 15 years of experience. Now we have added this information in section “Materials and Methods.” Specific characteristics for all subjects are provided in Table 1.

### Magnetic Resonance Imaging Data Acquisition

The MRI data from 2019 to 2021 were scanned preoperatively. Pre-surgery MRI images were acquired with a 3.0 GE Signa HDxt scanner with an 8-channel head-coil in the department of radiology, Yijishan Hospital of Wannan Medical College. High-resolution T1-weighted MR images were obtained by a 3D magnetization-prepared rapid gradient-echo (MPRAGE) with the following parameters: repeat time (TR) = 1900 milliseconds (ms), echo time (TE) = 2.49 ms, time inversion (TI) = 900 ms, matrix = 256 × 256, flip angle (FA) = 90°, thickness = 1 millimeter (mm), gap = 0.5 mm, slices = 176. Resting-state functional images, including 240 volumes, were obtained using a gradient-recalled echo-planar imaging (GRE-EPI) sequence, with TR = 2000 ms, TE = 30 ms, FA = 90°, acquisition matrix = 64 × 64, field of view (FOV) = 220 mm × 220 mm, thickness = 4.0 mm, gap = 0 mm, number of slices = 36, and voxel size = 3.4 mm × 3.4 mm × 4 mm.

### Image Pre-processing

Magnetic resonance imaging data were pre-processed using Matlab (Math Works Inc., Natick, MA, United States) and SPM8. The image processing procedure was conducted per published studies (Liu Y. et al., 2020; Chen et al., 2022). Briefly, the functional MRI (fMRI) image pre-processing steps included discarding the first 10 volumes, slice-timing, head motion corrections, spatial normalization, spatial smoothing, denoising, and temporal filtering (Liu Y. et al., 2020; Chen et al., 2022). A total of 2 non-FGep patents and 1 FGep patent were excluded from the subsequent analysis due to the excessive head movement (cumulative translation or rotation > 3.0 mm or 3.0°). We found no significant differences in head motion parameters between the groups (Power et al., 2012; Van Dijk et al., 2012).

During fMRI image pre-processing, the Diffeomorphic Anatomical Registration Through Exponentiated Lie Algebra (DARTEL) algorithm was used to normalize and segment the structural images into GM, WM, and cerebrospinal fluid (CSF) partitions (Ashburner and Friston, 2009). The native and DARTEL versions were used to compute total intracranial volumes (TIV). TIV was obtained by calculating the sum of GM, WM, and CSF tissues of all voxels in native space by using internal code. The structural and functional image processing procedure details are provided in the Supplementary Methods 1.

### Executive Control Network Functional Connectivity Analysis

Referring to previously published studies (Zhang et al., 2018), we made patients’ group GM mask without tumor area before performing FC analysis. Subsequent FC analysis was constrained within this GM mask. The details regarding the construction of group GM mask without tumor area are provided in the Supplementary Methods 2.

Several studies have agreed that the dorsolateral prefrontal cortex (DLPFC) is the critical brain region within the ECN (Seeley et al., 2007; Fransson and Marrelec, 2008; Smith et al., 2009). According to converging evidence (Seeley et al., 2007; Fransson and Marrelec, 2008; Smith et al., 2009; Dong et al., 2020; Liu et al., 2021), this study selected the right DLPFC (MNI space: 48, 12, 34) to draw 6-mm spheres as seed region of interest (ROI) (Seeley et al., 2007; Fransson and Marrelec, 2008; Smith et al., 2009; Dong et al., 2020; Liu et al., 2021). We performed a voxel-wise cross-correlation analysis between the individual averaged time series for all voxel within DLPFC and the whole brain within the patients’ group GM mask (Chen et al., 2016a,^2022^; Liu et al., 2021).

### Regional Homogeneity Analysis

As described in previous studies (Zang et al., 2004; Liu Y. et al., 2020), we measured ReHo to characterize the homogeneity of the time series in a local neighborhood of voxels within the ECN. The details of the ReHo analysis are provided in Supplementary Methods 3.

#### Pattern Classification Based on the Altered Gray Matter, Regional Homogeneity, and Functional Connectivity Within Executive Control Network

As described in previous studies (Chen et al., 2022), an support vector machine (SVM) approach was applied to test how well GM, ReHo, and FC within ECN could distinguish non-FGep and FGep from CN subjects. We performed a linear SVM classifier using the LIBSVM software.^1^ We assess the generalization of this SVM classifier and its accuracy, sensitivity, and specificity using a leave-one-out cross-validation (LOOCV) strategy. Accuracies obtained for each tested subject were averaged to obtain the LOOCV accuracy. The AUC was used to assess the classification power of the SVM classifier. The details of the LOOCV strategy are provided in Supplementary Methods 4.

### Statistical Analysis

#### Demographic, Neuropsychological Data, and Head Rotation Parameters

We performed a one-way analysis of variance (ANOVA) and chi-square test (only applied in the comparison of gender) to compare differences in demographic data (age, gender, and education level), cognitive measures, and head rotation parameters among CN, non-FGep, and FGep subjects (*p* < 0.05). For each neuropsychological test, the individual raw scores were transformed to *Z* scores to improve the statistical power for cognitive measures, according to the mean and standard deviation of the scores for all subjects. Notably, for tests measured in time (TMT), the raw scores were defined as the reciprocal of the time required for the test. *Post hoc* analysis was performed to investigate the source of ANOVA differences.

#### Group-Level Intrinsic Connectivity Analysis

To determine the ECN patterns for the CN, non-FGep, and FGep subjects, the FC spatial maps of each group were submitted to a random-effect analysis using a one-sample *t*-test after controlling for age, sex, education, and TIV. A stringent threshold of *p* < 0.001 was set using the permutation test with Threshold-Free Cluster Enhancement (TFCE) (Smith and Nichols, 2009) and the family wise error (FWE) correction for the whole brain to reveal regions that are most robustly correlated with each seed.

We used two-sample *t*-tests to assess the between-group differences of FCs of ECN after controlling for the effects of age, sex, education, and TIV. A stringent threshold of *p* < 0.001 was set using the permutation test with TFCE and the false discovery rate (FDR) correction.

#### Clinical Behavioral Significance of Altered Gray Matter, Regional Homogeneity, and Functional Connectivity

To assess the clinical behavioral significance of altered GM, ReHo, and FC, we extracted the values of the regions showing differential patterns among CN, non-FGep, and FGep subjects. We performed a correlation analysis to assess the relationships between the extracted GM, ReHo, and FC values and cognition after controlling for age, sex, and education. The significance level was set to *p* < 0.05 using FDR correction for multiple comparisons.

## Results

### Demographic and Neuropsychological Data

As shown in Table 1, no significant differences in age, gender, and education level were found between CN, non-FGep, and FGep groups (*p* > 0.05). The non-FGep showed significant deficits in information processing indicated by lower TMT scores. Similarly, FGep showed a substantial decrease in episodic memory (lower AVLT scores) and information processing speed (lower TMT scores) relative to CN (*p-values* < 0.05).

### Functional Connectivity Patterns and Inter-Group Differences Within the Executive Control Network

**FIGURE 1:**
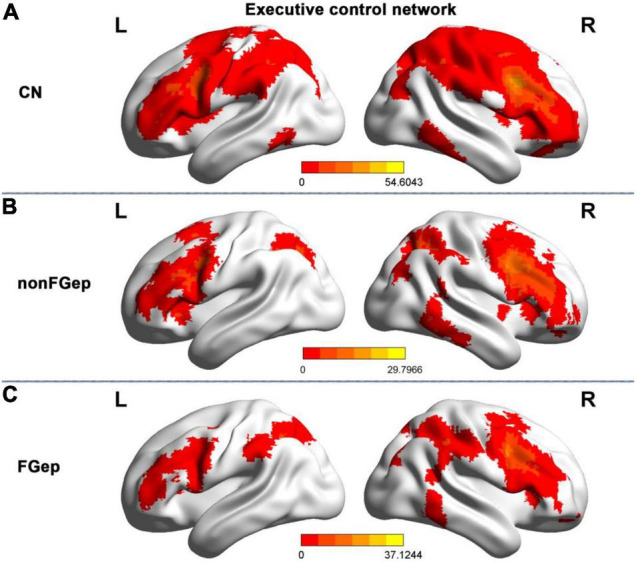
Resting-state functional connectivity patterns of the executive control network within group maps in CN **(A)**, non-FGep **(B)**, and FGep **(C)** subjects. CN, healthy controls; non-FGep, frontal glioma without epilepsy; FGep, frontal glioma with epilepsy; PreCG, Precentral gyrus; L, left hemisphere; R, right hemisphere.

**FIGURE 2:**
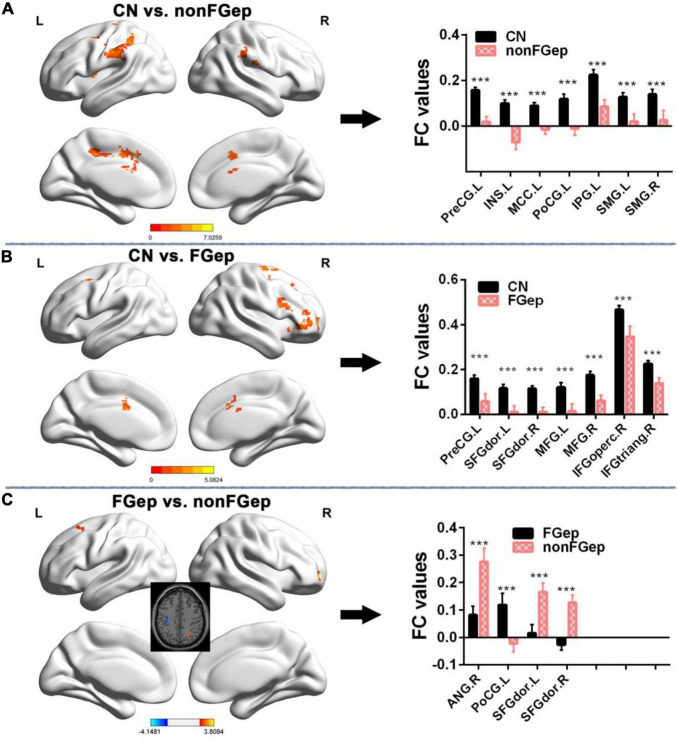
Comparison of FC within the executive control network between CN, non-FGep, and FGep subjects. **(A)** Altered regions of FC within the executive control network in non-FGep patients compared with CN. The bar chart on the right shows the quantitative comparison of FC in these altered regions. ***p < 0.001. **(B)** Altered regions of FC within the executive control network in FGep patients compared with CN. The bar chart on the right shows the quantitative comparison of FC in these altered regions. ***p < 0.001. **(C)** Different regions of FC within the executive control network between FGep and non-FGep patients. The bar chart on the right shows the quantitative comparison of FC in these different regions. ***p < 0.001. CN, healthy controls; non-FGep, frontal glioma without epilepsy; FGep, frontal glioma with epilepsy; L, left hemisphere; R, right hemisphere; PreCG, Precentral gyrus; INS, Insula; MCC, Middle Cingulate and paracingulate gyri; PoCG, Postcentral gyrus; IPG, Inferior parietal gyrus; SMG, Supramarginal gyrus; SFGdor, Superior frontal gyrus, dorsolateral; MFG, Middle frontal gyrus; IFGoperc, Inferior frontal gyrus, opercular part; IFGtriang, Inferior frontal gyrus, triangular part; ANG, Angular gyrus; FC, functional connectivity.

Functional connectivity patterns within the ECN are shown in Figure 1. CN, non-FGep, and FGep showed differential FC patterns within ECN (*p* < 0.001 corrected by TFCE-FWE). Compared with CN, non-FGep had reduced FC between the right DLPFC and left precentral gyrus (PreCG), insula (INS), middle cingulate and paracingulate gyri (MCC), postcentral gyrus (PoCG), inferior parietal gyrus (IPG), and bilateral supramarginal gyrus (SMG) within the ECN (Figure 2A and Table 2, *p* < 0.001 corrected by TFCE-FDR). The FGep group had decreased FC between the right DLPFC and left PreCG, bilateral superior frontal gyrus (SFGdor), bilateral middle frontal gyrus (MFG), right inferior frontal gyrus, opercular part (IFGoperc), right inferior frontal gyrus, and triangular part (IFGtriang) within the ECN, compared to CN (Figure 2B, *p* < 0.001 corrected by TFCE-FDR). Comparing non-FGep and FGep, within the ECN, FGep had diminished FC between the right DLPFC and the right ANG and bilateral SFGdor, and increased FC between the right DLPFC and left PoCG (Figure 2C, *p* < 0.001 corrected by TFCE-FDR).

**TABLE 2:** Comparison of GM, ReHo, and FC of ECN between CN, non-FGep, and FGep subjects.

Brain regions	L/R	MNI			*T-*values	Cluster size (mm^3^)
		x	y	z		
**Functional connectivity of ECN**						
**CN vs. Non-FGep**						
PreCG	L	39	4	16	5.901	3591
INS	L	−36	3	12	6.0199	1242
MCC	L	−15	−36	39	4.8353	2403
PoCG	L	−36	−15	42	3.9927	2781
IPG	L	−39	−44	49	4.2246	7425
SMG	L	−57	−27	36	3.8981	2673
SMG	R	51	−18	24	4.5986	3240
**CN vs. FGep**						
PreCG	L	−33	−9	45	5.0824	2295
SFGdor	L	−33	0	66	3.6972	1215
SFGdor	R	18	0	50	3.5153	4968
MFG	L	−27	−3	48	3.6	2430
MFG	R	27	51	3	3.1439	3942
IFGoperc	R	39	3	24	3.5776	1593
IFGtriang	R	42	27	0	3.5805	3186
**Non-FGep vs. FGep**						
ANG	R	30	−60	36	3.8094	783
PoCG	L	−33	−21	42	−3.5288	702
SFGdor	L	−18	21	57	3.2738	1080
SFGdor	R	27	57	−3	3.7863	1053
**GM**						
**CN vs. Non-FGep**						
IPG	L	−38	−44	50	−5.6639	2336
PoCG	L	−64	−16	34	−4.5346	464
**CN vs. FGep**						
SFGdor	R	14	0	76	−6.5057	528
PreCG	L	−30	8	64	−4.8559	400
**Non-FGep vs. FGep**						
None						
**ReHo**						
**CN vs. Non-FGep**						
INS	L	−24	−3	9	−9.7065	2673
PreCG	L	−33	−12	39	−6.1399	2052
**CN vs. FGep**						
None						
**Non-FGep vs. FGep**						
None						

*CN, healthy controls; non-FGep, frontal glioma without epilepsy; FGep, frontal glioma with epilepsy; PreCG, Precentral gyrus; INS, Insula; MCC, Middle Cingulate and paracingulate gyri; PoCG, Postcentral gyrus; IPG, Inferior parietal gyrus; SMG, Supramarginal gyrus; SFGdor, Superior frontal gyrus, dorsolateral; MFG, Middle frontal gyrus; IFGoperc, Inferior frontal gyrus, opercular part; IFGtriang, Inferior frontal gyrus, triangular part; ANG, Angular gyrus; GM, gray matter; ReHo, regional homogeneity. MNI, Montreal neurological institute.*

### Differences in Gray Matter and Regional Homogeneity Within the Executive Control Network

**FIGURE 3:**
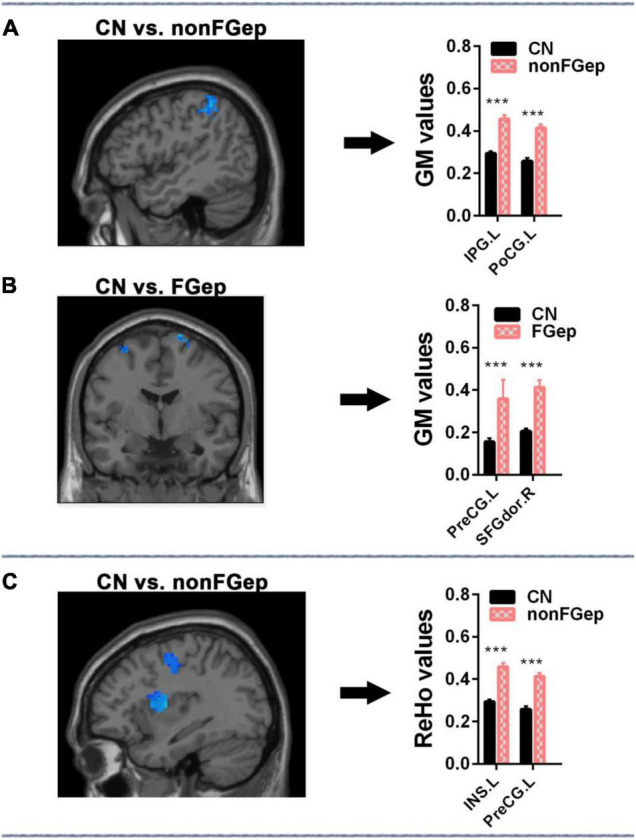
Comparison of ReHo and GM within the executive control network among CN, non-FGep, and FGep subjects. **(A)** Altered regions of GM within the executive control network in non-FGep patients compared with CN. The bar chart on the right shows the quantitative comparison of GM in these altered regions. ***p < 0.001. **(B)** Altered regions of GM within the executive control network in FGep patients compared with CN. The bar chart on the right shows the quantitative comparison of FC in these altered regions. ***p < 0.001. **(C)** Altered regions of ReHo within the executive control network in non-FGep patients compared to CN. The bar chart on the right shows the quantitative comparison of ReHo in these different regions. ***p < 0.001. CN, healthy controls; non-FGep, frontal glioma without epilepsy; FGep, frontal glioma with epilepsy; PreCG, Precentral gyrus; INS, Insula; PoCG, Postcentral gyrus; IPG, Inferior parietal gyrus; SFGdor, Superior frontal gyrus, dorsolateral; GM, gray matter; ReHo, regional homogeneity.

Compared with CN, non-FGep showed increased GM in left PoCG and left IPG (Figure 3A and Table 2, *p* < 0.05 corrected by FDR) and increased ReHo in left INS and left PreCG (Figure 3C and Table 2, *p* < 0.05 corrected by FDR). The FGep showed increased GM in left PreCG and right SFGdor than CN (Figure 3B and Table 2, *p* < 0.05 corrected by FDR).

### Clinical Behavioral Significance of Altered Gray Matter, Regional Homogeneity, and Functional Connectivity

**FIGURE 4:**
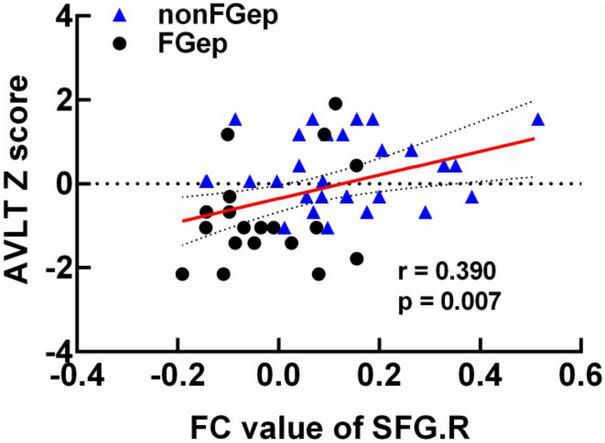
Clinical behavioral significance of brain regions with differential FC between non-FGep and FGep subjects. non-FGep, frontal glioma without epilepsy; FGep, frontal glioma with epilepsy; SFG, superior frontal gyrus; FC, functional connectivity; AVLT, Auditory Verbal Learning Test- 20-min delayed recall.

The correlation analysis demonstrated that FC between right DLPFC and left PoCG within the ECN was positively correlated with the AVLT score in FGep and non-FGep (Figure 4, *r* = 0.390, *p* = 0.007, FDR correction). Moreover, no significant correlations were found between GM, ReHo, FC, and other cognitive functions (*p*-values < 0.05).

### Classification of CN, Non-FGep, and FGep Patients Based on the Altered Gray Matter Volumes, Regional Homogeneity, and Functional Connectivity

**FIGURE 5:**
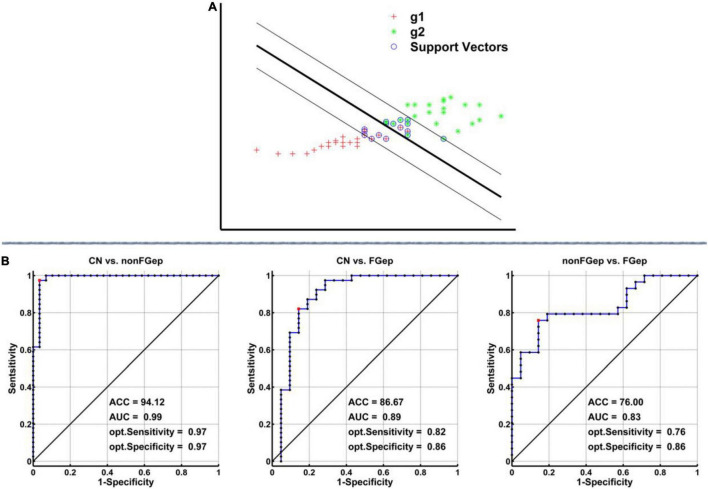
Classification power of the MRI-based “classifier” in distinguishing individuals as non-FGep vs. CN, FGep vs. CN, and non-FGep vs. FGep. **(A)** The schematic diagram indicated a support vector machine “classifier” based on altered structural (GM) and functional (ReHo and FC) characteristics within the executive control network. **(B)** ROC curve showing the classification power of the MRI-based “classifier.” The values of ACC, AUC, sensitivity, and specificity are marked in the lower right of the figure. CN, healthy controls; non-FGep, frontal glioma without epilepsy; FGep, frontal glioma with epilepsy; ACC, accuracy; AUC, area under the curve; GM, gray matter; ReHo, regional homogeneity; FC, functional connectivity.

As shown in Figure 5, the SVM classifier’s classification accuracy was 94.12% for non-FGep from CN, 86.67% for FGep from CN, and 76% for FGep from non-FGep. The ROC curve of the SVM classifier showed a high power for distinguishing non-FGep from CN, FGep from CN, and FGep from non-FGep on an individual subject basis, with high AUC (99.0, 89.0, and 83.0%, respectively), sensitivity (97.0, 82.0, and 76.0%, respectively), and specificity (97.0, 86.0, and 86.0%, respectively).

## Discussion

To our best knowledge, this was the first study to evaluate structural and functional abnormalities of ECN in patients with frontal glioma-related epilepsy and to investigate whether the abnormalities can accurately identify glioma-related seizures. Three main results of the study should be emphasized. First, compared with CN, non-FGep and FGep showed convergent and divergent altered patterns of ECN based on the structural and functional level of the brain. Second, FC between right SFG and right DLPFC was positively correlated with the AVLT scores in non-FGep and FGep. Lastly, the SVM classifier based on structural and functional abnormalities within the ECN could accurately differentiate non-FGep and FGep from CN, and FGep from non-FGep on an individual basis with very high accuracy, AUC, sensitivity, and specificity. These findings indicate that specific functional changes can assist in the early identification and intervention of epileptic seizures in frontal glioma patients.

This study found that non-FGep had reduced FC within ECN, mainly distributed in PreCG, INS, MCC, PoCG, IPG, and SMG. FGep also showed decreased FC within ECN, distributed primarily in PreCG, SFGdor, MFG, IFGoperc, and IFGtriang. These results suggest that non-FGep and FGep showed convergent and divergent altered patterns of ECN. These ECN network findings are supported by behavioral results with significantly lower executive function scores (TMT scores) in both non-FGep and FGep. Our results are inconsistent with previous studies (Liu et al., 2019; Liu Y. et al., 2020; Tordjman et al., 2021), which showed that frontal glioma patients have the clinical compensation phenomenon of intact executive function and increased FC within ECN. Their interpretation is that tumor invasion induces FC reorganization of the ECN. This inconsistency could be because compensation and decompensation coexist dynamically in disease progression, and our study subjects may have been in the decompensation stage (Belleville et al., 2011; Chen et al., 2015, 2016a; Liu Y. et al., 2020). Another reason could be that the method for driving FC analysis of ECN in this study is inconsistent with their approach. Our research adopts a seed-based, data-driven approach in the whole brain for calculating FC. In contrast, their research used an independent component analysis and FC between regions within the ECN mask. Furthermore, our findings suggest that tumor invasion in non-FGep was more likely to diminish FC in remote brain regions, while tumor invasion in FGep was more likely to decrease FC in nearby brain regions. These findings support the idea that tumor invasion of cortical lesions leads to the disruption of connections between local and remote brain regions in the form of functional networks (Vigneau et al., 2006; Carter et al., 2010; Xiang et al., 2010; Briganti et al., 2012).

Notably, FGep showed decreased FC in ANG, PoCG, and SFG.dor within ECN compared to non-FGep and CN. Contrastingly, non-FGep showed no difference in FC in SFG.dor. FC between right SFG and right DLPFC was also positively correlated with AVLT scores in non-FGep and FGep. These findings suggest that tumor invasion of the frontal lobe accompanied by epileptic seizures exacerbates functional abnormalities within ECN, and the reduced FC contributes to episodic memory impairment in FGep. Clinical behavioral results support this theory, wherein non-FGep showed intact episodic memory indicated by AVLT scores, while FGep showed significant episodic memory impairment. Indeed, previous studies have confirmed the interaction between the ECN and the episodic memory network with the frontal lobe as the interactive hub (Yuan et al., 2016; Xu et al., 2020). Therefore, we propose that the frontal lobe (SFG) may be an early neuroimaging marker of episodic memory decline during epileptic episodes in patients with frontal glioma.

Another unique contribution of this study is that tumor invasion of the frontal lobe induced local structural (GM volume) and functional (ReHo) reorganization only, regardless of an epileptic seizure. These results align with previous studies that found that tumor invasion induces the brain’s structural and functional reorganization (Liu et al., 2019; Hu et al., 2020; Liu D. et al., 2020; Liu Y. et al., 2020). However, unlike these studies, our results found reorganization occurred at perilesional and remote recruitment (Duffau, 2014; Almairac et al., 2018). It is also possible that different isocitrate dehydrogenase 1 (IDH1) mutation statuses lead to different patterns of network reorganization (Qi et al., 2022). This study did not identify the IDH1 mutation status. Interestingly, reorganization of tumor invasion with seizure occurred in perilesional recruitment, whereas reorganization without seizure occurred in remote recruitment. This suggests that the seizures may have more to do with the tumor itself. Therefore, seizure-induced brain reorganization is more likely to occur around the lesion.

This study also showed that non-FGep and FGep showed compensatory activity in the same brain region: PreCG. The PreCG is associated with sensorimotor and visuo-spatial functions (Davion et al., 2021; Li et al., 2022). Our behavioral results showed that visuospatial cognitive function was intact in both groups. Therefore, we suggest that tumor invasion induced structural and functional reorganization of the PreCG compensated for the visuospatial cognitive function of the patients. Although the SFG.dor was compensated in FGep, its FC decreased significantly, and episodic memory was impaired. This indicates that seizures in glioma patients may accelerate the progression from the compensatory to the decompensated stage.

Our most fascinating finding was that by combining structural and functional abnormalities within the ECN, the SVM classifier could accurately differentiate non-FGep and FGep from CN and FGep from non-FGep on an individual basis with very high accuracy, AUC, sensitivity, and specificity. Structural and functional features within ECN may reflect epilepsy susceptibility in patients with frontal glioma and can effectively detect frontal glioma-related epilepsy. Although, in recent years, some studies have constructed prediction models of epileptic seizures in glioma by using radiomic features and ML (Liu et al., 2018; Gao et al., 2021), they only use the information of segmented and masked tumor lesions. These models have some limitations. First, there are many manual factors in the segmentation and selection of tumors. Second, most glioma-related seizures are not located in the lesion but the peripheral or remote brain region. The radiomic features based on tumor lesions cannot truly reflect the characteristics of epileptic seizures.

Furthermore, some studies suggest that tumor invasion affects brain function over the whole network rather than isolated regions (Vigneau et al., 2006; Xiang et al., 2010; Briganti et al., 2012). In particular, some studies indicated that tumor-related epilepsy induces topological changes in WM networks (Zhou et al., 2022) and functional networks (Fang et al., 2022). Therefore, the network-based features and the structural features outside the tumor lesion can reflect more information about glioma-related epilepsy. The effective neuroimaging features will be highly conducive to the prediction of seizures. In addition, radiomic analysis can incorporate too many features in a small sample of data, which can easily result in over-fitting or bias of the prediction model. Our study included the structural and functional abnormalities within the ECN that have the highest association with the impact of tumor invasion of the frontal lobe as features of the model, which improved the predictive power and generalization of the model.

However, this study has certain limitations: First, a relatively small sample size, especially for patients with glioma with seizures (*n* = 21). Larger samples will be needed in the future to verify our results. Second, we only evaluated the GM volume, and future studies need more indicators to assess the structure, such as fiber integrity measured by diffusion tensor technology. Finally, this study was a single-center study that could not evaluate the model’s generalization. The stability of the model needs to be verified in independent samples. Future multi-center studies are needed to verify the generalization of our model in distinguishing glioma epileptic seizures.

## Conclusion

Tumor invasion of the frontal lobe induces local structural and functional reorganization within the ECN. These structural and functional abnormalities are exacerbated by accompanying epileptic seizures. ECN abnormalities, which are related to cognitive impairment, can be used to accurately distinguish the presence or absence of epileptic seizures in patients with frontal glioma at an early stage. Our findings raise the possibility that specific functional changes can assist in the early identification and intervention of epileptic seizures in frontal glioma patients.

## Data Availability Statement

The raw data supporting the conclusions of this article will be made available by the authors, without undue reservation.

## Ethics Statement

The studies involving human participants were reviewed and approved by the Human Participants Ethics Committee of Yijishan Hospital of Wannan Medical College (China). The patients/participants provided their written informed consent to participate in this study.

## Author Contributions

GD and XJ designed the study. MT, RX, WZ, KD, and ZH collected the data. GD, RX, XC, and HZ analyzed the data and prepared the manuscript. All authors read and approved the final manuscript.

## Conflict of Interest

The authors declare that the research was conducted in the absence of any commercial or financial relationships that could be construed as a potential conflict of interest.

## Publisher’s Note

All claims expressed in this article are solely those of the authors and do not necessarily represent those of their affiliated organizations, or those of the publisher, the editors and the reviewers. Any product that may be evaluated in this article, or claim that may be made by its manufacturer, is not guaranteed or endorsed by the publisher.

## Footnotes

1
https://www.csie.ntu.edu.tw/∼cjlin/libsvm/
