# Supplementary material for: Altered Structural and Functional Patterns Within Executive Control Network Distinguish Frontal Glioma-Related Epilepsy
Source: Front Neurosci. 2022 May 26;16:916771. doi: 10.3389/fnins.2022.916771 (PMC9179179; doi:10.3389/fnins.2022.916771)
Supplement: Supplementary file 1 [file Data_Sheet_1.pdf]

Altered structural and functional patterns within executive control network distinguish frontal glioma-related epilepsy

Supporting Information

SI methods

S.1 Image preprocessing analysis
S.2 Construction of group grey matter mask without tumor area
S.3 Regional homogeneity (ReHo) analysis
S.4 Pattern classification based on the altered GM, ReHo, and FC within ECN

Supplementary figure legends

Figure S1 Relationships between fitness curve accuracy and iteration number for machine learning

Supporting Information

SI methods

S.1 Image preprocessing analysis

We used MATLAB2015a (http://www.mathworks.com/products/matlab/) and DPABI to preprocess the MRI data (Yan et al., 2016). Firstly, we discarded the first ten images to minimize effects of scanner signal stabilization. Then we omitted scans with head motion exceeding 3mm or 3°of maximum rotation through the resting -state run. Then we calculated framewise displacement (FD) for all resting state volumes after realigning, slice timing correction, and co-registration (Power et al., 2012). Functional and structural images were co-registered. Structural images were then normalized and segmented into gray matter, white matter and cerebrospinal fluid signal (CSF) partitions using the DARTEL technique. The realigned fMRI data were normalized by using the EPI template into the standard MNI space and resampled to an isotropic voxel size of 3 mm, and then smoothed by a Gaussian kernel of 6 mm^3 full-width. Nuisance covarites regression including Friston 24-parameter model: 6 head motion parameters, 6 head motion parameters one time point before, and the 12 corresponding squared items (Friston et al., 1996), CSF, white matter, and the global signals as well as the linear trend were created and removed using partial regression with scrubbing (Power et al., 2014; Yan et al., 2013). After nuisance covariate regression, the resultant data were band pass filtered to select low frequency (0.01-0.1Hz) signals (Liu et al., 2019; Liu et al., 2020). Voxels within a group derived gray matter mask were used for further analyses.

S.2 Construction of group grey matter mask without tumor area

Referring to previous studies (Zhang et al., 2018), two senior neurosurgeons used MRIcroN (http://people.cas.sc.edu/rorden/mricron.html) to manually trace tumors on individual 3D structural images: T1 enhancement images for gliomas with enhancement, and T2-flair images coregistered to T1 weighted images for gliomas without enhancement. The third senior neurosurgeon confirmed the accuracy of the manual tracing.Then a tumor mask for each patient was created after manually tracing the tumor. This tumor mask is spatially normalized to the standard MNI template, and we binarized and tacked all patients' tumor masks together to construct a tumor overlapping region. We further calculated the intersection of this tumor overlapping region and the gray matter mask (obtained by thresholding the gray matter probability map in SPM12 with probability larger than 0.2). Then we use this gray matter mask to reduce the intersection mask to obtain the final patients' group gray matter mask. Subsequent functional connectivity analysis was restricted to the non-tumor gray matter regions.

S.3 Regional homogeneity (ReHo) analysis

Refered to previously published studies (Liu et al., 2020; Zang et al., 2004), we measured ReHo to characterize similarity or homogeneity of the time series in a local neighborhood of voxels within ECN. ReHo maps were generated by calculating Kendall's coefficient concordance (KCC) of the fMRI time series of a specific voxel with all adjacent voxels (this model can be seen as a 3×3×3 cube). ReHo ranges from 0 to 1, with the higher values indicating greater similarity of time series in the local neighborhood. The ReHo map was then standardized by dividing the KCC of each voxel by the average KCC of the entire brain. Finally, regional estimates were calculated for each

subject by averaging the Z-scores of the voxels.

S.4 Pattern classification based on the altered GM, ReHo, and FC within ECN

Refered to previously published studies (Chen et al., 2022), a SVM approach was applied to test how well GM, ReHo, and FC within ECN could distinguish non-FGep and FGep from CN subjects. We performed a linear SVM classifier using LIBSVM software (Software available at http://www.csie.ntu.edu.tw/˜cjlin/libsvm). We assess the generalization of this SVM classifier and its accuracy, sensitivity, and specificity using a leave-one-out cross-validation (LOOCV) strategy. We averaged all accuracies obtained at each tested subject to obtain the LOOCV accuracy. Briefly, if there are N samples in total, in each LOOCV experiment, the N-1 samples are viewed as the training set, and the omitted one is used as a test subject to computing the classification error.

Supplementary figures and legends

Figure S1 Relationships between fitness curve accuracy and iteration number for machine learning

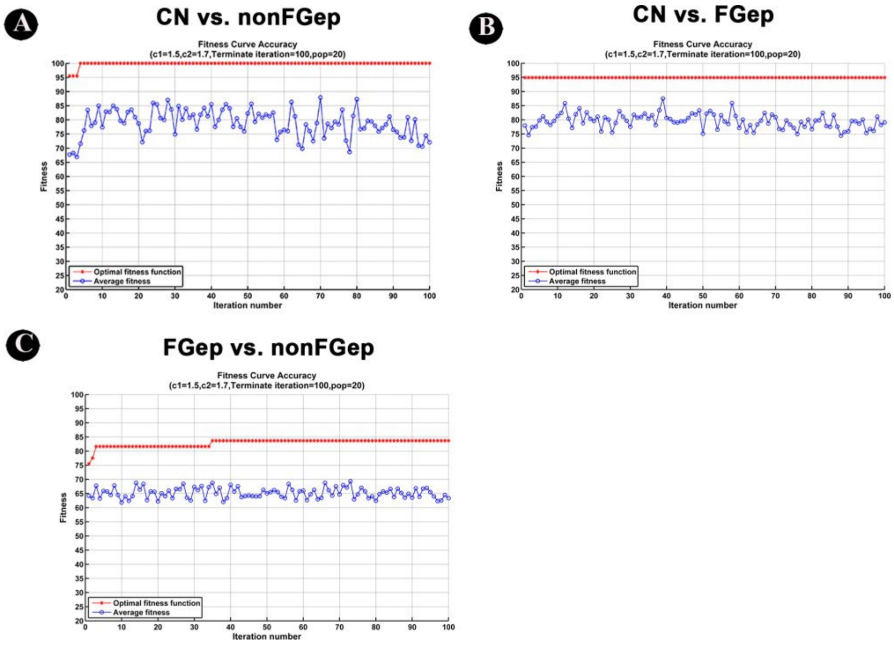

Abbreviations: CN, healthy controls; non-FGep, frontal glioma without epilepsy; FGep, frontal glioma with epilepsy.

References

Chen, J., Chen, R., Xue, C., Qi, W., Hu, G., Xu, W., Chen, S., Rao, J., Zhang, F., Zhang, X., 2022. Hippocampal-Subregion Mechanisms of Repetitive Transcranial Magnetic Stimulation Causally Associated with Amelioration of Episodic Memory in Amnestic Mild Cognitive Impairment. J Alzheimers Dis. 85, 1329-1342.

Friston, K.J., Williams, S., Howard, R., Frackowiak, R.S., Turner, R., 1996. Movement-related effects in fMRI time-series. Magn Reson Med. 35, 346-55.

Liu, D., Hu, X., Liu, Y., Yang, K., Xiao, C., Hu, J., Li, Z., Zou, Y., Chen, J., Liu, H., 2019. Potential Intra- or Cross-Network Functional Reorganization of the Triple Unifying Networks in Patients with Frontal Glioma. World Neurosurg.

Liu, Y., Hu, G., Yu, Y., Jiang, Z., Yang, K., Hu, X., Li, Z., Liu, D., Zou, Y., Liu, H., Chen, J., 2020.

Structural and Functional Reorganization Within Cognitive Control Network Associated With Protection of Executive Function in Patients With Unilateral Frontal Gliomas. Front Oncol. 10, 794.

Power, J.D., Barnes, K.A., Snyder, A.Z., Schlaggar, B.L., Petersen, S.E., 2012. Spurious but systematic correlations in functional connectivity MRI networks arise from subject motion. Neuroimage. 59, 2142-54.

Power, J.D., Mitra, A., Laumann, T.O., Snyder, A.Z., Schlaggar, B.L., Petersen, S.E., 2014. Methods to detect, characterize, and remove motion artifact in resting state fMRI. Neuroimage. 84, 320-41.

Yan, C.G., Craddock, R.C., Zuo, X.N., Zang, Y.F., Milham, M.P., 2013. Standardizing the intrinsic brain: towards robust measurement of inter-individual variation in 1000 functional connectomes. Neuroimage. 80, 246-62.

Yan, C.G., Wang, X.D., Zuo, X.N., Zang, Y.F., 2016. DPABI: Data Processing & Analysis for (Resting-State) Brain Imaging. Neuroinformatics. 14, 339-51.

Zang, Y., Jiang, T., Lu, Y., He, Y., Tian, L., 2004. Regional homogeneity approach to fMRI data analysis. Neuroimage. 22, 394-400.

Zhang, N., Xia, M., Qiu, T., Wang, X., Lin, C.P., Guo, Q., Lu, J., Wu, Q., Zhuang, D., Yu, Z., Gong, F., Farrukh Hameed, N.U., He, Y., Wu, J., Zhou, L., 2018. Reorganization of cerebro-cerebellar circuit in patients with left hemispheric gliomas involving language network: A combined structural and resting-state functional MRI study. Hum Brain Mapp. 39, 4802-4819.
